# A Mediation Model on How Conspiracy Beliefs Concerning the Corona-Crisis Are Related to Corona-Related Behaviours

**DOI:** 10.3389/fpsyg.2021.740888

**Published:** 2021-11-22

**Authors:** Arie Dijkstra

**Affiliations:** Department of Social Psychology, University of Groningen, Groningen, Netherlands

**Keywords:** COVID-19, conspiracy beliefs, psychological determinants, prevention behaviours, testing behaviour, getting vaccinated

## Abstract

**Background:** The endorsement of Conspiracy Beliefs concerning corona (CBc) may make people reject information from the general media, leading them to not follow recommendations on prevention behaviours, getting tested, and getting vaccinated. The aim of the present study was to understand the relationship between CBc and engaging in these corona-related behaviours.

**Method:** Two samples of participants (*N* = 1,004 and *N* = 159) were recruited independently. Participants filled in a survey that assessed four indicators of the three behaviours, four general psychological determinants (e.g., the seriousness of COVID-19), five behaviour-specific psychological determinants (e.g., test reliability, vaccine effectiveness), and CBc.

**Results:** The explained variances of the different models with regard to the four indicators of behaviour ranged from 3.9 to 75%. Mediation analyses using Hayes PROCESS model 4 showed significant mediation by general determinants in both samples, and mediation by several behaviour-specific determinants in one sample.

**Discussion:** Conspiracy Beliefs concerning corona may lead to rejection of general media information, and this may lead to states of psychological determinants that do not stimulate to engage in prevention behaviours, testing, or vaccination. The present study shedS some light on how CBc could be related to corona-related behaviours.

## Introduction

The thinking and behaviour of individuals are essential determinants of the pandemic (Drinkwater et al., 2021): When all people would engage optimally in all the recommended behaviours, the pandemic would be stopped soon. Especially, three (clusters of) behaviours comprise an important angle to control and stop the pandemic: prevention behaviours (e.g., hand washing, keeping distance), getting tested, and getting vaccinated. Despite exposure to largely the same general media in the Netherlands (i.e., national and regional TV, radio and newspapers, and their internet pages), there is substantial variance in how people think and behave in relation to COVID-19. One psychological factor that has recently been found to be related to the above corona-related behaviours is conspiracy beliefs concerning the corona-crisis. The aim of the present study was to understand how, in the context of the role of the general media in the present corona-crisis, these conspiracy beliefs might determine these three behaviours.

Conspiracy beliefs are manifestations of conspiracy theories, which are “attempts to explain the ultimate causes of significant social and political events and circumstances with claims of secret plots by two or more powerful actors” (Douglas et al., 2019, p. 3). The literature identifies several conspiracy theories that are endorsed by groups of people, for example, about the death of former United States President John F. Kennedy (Enders and Smallpage, 2018), about the death of Princess Diana (Douglas and Sutton, 2008), and the existence of a secret world elite that abuses children (Bloom and Moskalenko, 2021). The common ground of the theories is that governments or other powerful organisations are responsible for serious, negative happenings. The theories imply that these happenings are deliberately and covertly orchestrated by governments or their institutions or other powerful organisations for hidden purposes, for example, to control people or to benefit financially. For further reading about conspiracy theories, their causes, and their effects, we refer to the review of Douglas et al. (2019). Recently, conspiracy beliefs about the present corona-crisis have been identified.

Conspiracy Beliefs about the present corona-crisis (CBc) refer to several conspiracy theories (Lewis, 2020). The recent literature review of van Mulukom et al. (2021) provides a valuable overview. For example, the COVID-19 pandemic might be a hoax or a bioweapon which is spread through misinformation or 5G networks by China or by companies for financial gain or to create a new world order. This review also shows that CBc can be related to the above corona-pandemic-related behaviours. For example, endorsement of CBc was related negatively to the intention for social distancing (Biddlestone et al., 2020; Bierwiaczonek et al., 2020), and the intention to get vaccinated (Romer and Jamieson, 2020).

Although the relation of CBc with corona-related behaviours seems robust, few data and theories are available on how this relationship develops; it is not immediately clear how CBc can determine corona-related behaviours. The causes of behaviours such as prevention behaviours, getting tested, and getting vaccinated, are normally explained by social cognitive determinant models (Bandura, 1986; Hagger et al., 2020); these behaviours are caused by psychological determinants. The present study tested whether the relationship between CBc and corona-related behaviours is mediated by the psychological determinants. No studies have assessed mediation of CBc using a full model of determinants, although relevant mediation findings have been published. For example, in another field of investigation, Jolley and Douglas (2014a) showed that exposure to conspiracy theories was related to lower intentions to engage in politics and to behave pro-environmentally and that this relation was mediated by perceptions of powerlessness toward the government. Marinthe et al. (2020) showed that the relationship between conspiracy mentality and corona prevention behaviours was mediated by risk perception. Romer and Jamieson (2020) found that the relationship between conspiracy beliefs regarding corona and taking preventive action was mediated by the experience of a national threat, and between conspiracy beliefs and vaccination intention was mediated by the expected harm done by the vaccination. The present study will test mediation by a full model of psychological determinants of three behaviours; preventive actions, getting tested, and getting vaccinated. Below a brief outline of these common social-cognitive determinants will be given, with the distinction between general and behaviour-specific determinants.

General determinants of corona-related behaviours are people’s perceptions regarding COVID-19. These perceptions provide the reasons to engage in any behaviour that might lower the risk of getting ill from COVID-19. The Extended Parallel Process Model (EPPM; Witte, 1992) maps how persuasive health messages designed to prevent specific illnesses (e.g., skin cancer due to sun exposure) are processed, and can lead to adequate behaviour change or rejection of the message. The EPPM conceptualises a perceived threat as the main general psychological and motivational cause of any behaviour that can avert the threat. This threat can manifest as fear, and it is related to the underlying perceptions of the seriousness of the disease (COVID-19), and susceptibility to the disease (the risk of contracting COVID-19). Besides these threat-related general factors, the EPPM conceptualizes a control-related factor and efficacy. In the present study, this was operationalized as a general estimation of control over the outcome (COVID-19), leaving unspecified how the control would be exerted. Thus, seriousness, susceptibility, fear, and control are general potential psychological causes of corona-related behaviours.

Besides the above general psychological factors, behaviours are also governed by more behaviour-specific psychological factors. This notion is stressed in the most used behavioural model, the Theory of Planned Behaviour (TPB; Ajzen, 1991): Specific behaviours are caused by specific psychological causes. The TPB is a generic model of behavioural determinants that has been applied to various behaviours, including health behaviours. In the TPB, attitudinal beliefs about the effectiveness of investing in a specific behaviour are important: “Does the specific behaviour lead to the desired effect?”. With regard to prevention behaviours the specific belief refers to the effectiveness of the specific behaviour in preventing virus transmission. With regard to getting tested, a core-specific belief is an estimation of whether the test results are reliable. With regard to vaccination, a core-specific belief is the estimation of the effectiveness of the vaccination to prevent COVID-19.

An additional psychological determinant was assessed that is congruent with the TPB; moral norm (Rivis et al., 2009). As corona-related behaviours also have consequences for others, not only for oneself, the behaviours gain a social meaning (Young and Goldstein, 2021). Moral norms are people’s perceptions about the social desirability of a specific behaviour; whether a behaviour is socially correct or incorrect. People may differ in the extent to which they experience that engaging in, for example, COVID-19 testing or vaccination, is their social obligation or social duty. When this perception is internalized, it may also operate as a personal norm (Parker et al., 1995). A stronger sense of social duty may determine people to adhere more strongly to the recommended corona-related behaviours. In sum, CBc is thought to lead to changes in these general and behaviour-specific psychological determinants which, in turn, will lead to behaviour.

As an interpretational framework, CBc influence how people make sense of the world around them (Higgins, 1996; Greifeneder et al., 2004). CBc may influence how people interpret information about the world they are exposed to. In the Netherlands, one important source of information on the corona-crisis are the general media. When people think that the general media are involved in some conspiracy on the level of government and its organisations, they may receive the information as unreliable. That is, perceptions of conspiracies lower the trust in authorities and governments (Jolley and Douglas, 2014a; Douglas et al., 2019), which may undermine them as credible sources of information (Pornpitakpan, 2004), as the content and the selection of information may possibly be in function of the goals of the conspiracy rather than being authentic (Hannah, 2021). Also, with regard to the corona-crisis, studies show the relationship between CBc and lowered trust in authorities (Pummerer et al., 2021; van Mulukom et al., 2021). Similarly, information from others who are (largely) informed by the general media or communicate the same messages as the general media may also be received as unreliable as the information origins from the same incredible sources. Indeed, endorsement of conspiracy theories is related to interpersonal distrust (Goertzel, 1994). In addition, conspiracy theories (Jolley et al., 2019) and CBc (Marinthe et al., 2020) are related to non-normative or anti-social behaviours.

In the present theorizing, people who endorse CBc understand the information about COVID-19 from the media and from others through the lens of their CBc. In other words, they give meaning to these messages from the general media against the background of the CBc. This is in line with the Unimodel (Kruglanski et al., 2006), in which the persuasive effects of information on people crucially depend on the “major premises” people use to judge the information. For example, a governmental website X may communicate the argument “Because COVID-19 is a serious illness, people should comply to the recommended prevention behaviours.” If a person endorses the major premise “Media are part of a conspiracy and therefore are unreliable” together with the minor premise “Website X is part of the conspiracy,” the person will reject the information. Thus, from the Unimodel point of view, CBc may work as a major premise. Importantly, from this perspective “…evidence is in the mind of the beholder” (p. S110; Kruglanski et al., 2006). This perspective is consistent with Douglas et al.’s (2019) notion that “…the level of influence [of CT] appears to depend on pre-existing attitudes” (p. 18). The CBc-interpretation of the corona-related information will lead to rejection of the common claims communicated through the media about COVID-19 being a serious illness, about the contagiousness of mutations, the effectiveness of mouth caps, the reliability of tests, and the effectiveness of a vaccine, and more.

The aim of the present study was to understand how CBc might determine corona-related behaviours in the context of the role of the general media in the present corona-crisis. This study was conducted in the Dutch population, in which most people are exposed to the general media (television, radio, newspapers, news websites) that provide common knowledge on the corona-crisis and COVID-19 and communicate that COVID-19 is a serious disease that warrants behavioural actions. Possibly different from the media landscape in the United States (Romer and Jamieson, 2020), there is no strong distinction between more liberal or more conservative general media. People do have access to all kinds of social media that might propagate messages that oppose those of the general media. But a survey at the start of the pandemic in the Netherlands shows that people most often got their information on corona-related issues from the general (traditional) media, such as radio, TV, newspapers, and their websites (University of Amsterdam, 2021, September 5). Eight months and two corona-waves later a representative survey shows again that people still use those general media as their major source of information, and that they have become more suspicious of social media (van der Veer, 2021, September 5). In addition, the general media in the Netherlands seem to provide a rather similar perspective on the corona-pandemic (see DaarDan, 2020, June). This media situation is the context in which the present study was conducted.

Two samples of Dutch citizens were recruited through different channels (*N* = 1,004 and *N* = 159). Both samples answered the same questions in an online survey during the third corona-wave in the Netherlands in 2021. The research question is: To what extend is the relationship of CBc with corona-related behaviours (prevention, testing, vaccination) mediated through the determinants of behaviour.

## Materials and Methods

### Recruitment

To increase insight into the reliability of the findings, data from identical measurements in two samples from the general population of 16 years and older were gathered. The samples were recruited through different channels in the midst of the third corona wave in the Netherlands.

Sample 1 participants were recruited by a professional research company Enigma Research^1^ that hosts a panel of people in the three Northern provinces of the Netherlands. Their default procedure was used to select and pay participants. The data were gathered in the electronic survey system of the organisation. Participants filled in the survey in the first 2 weeks of April 2021.

Sample 2 participants were recruited by a snowball technique that started at the Facebook and WhatsApp accounts of the author in 2020, in which a first measurement was conducted that is not be reported here. The participants who indicated to be willing to join a later study were asked to leave their email addresses. These participants were contacted again by email for the present study. The link they were sent brought them to the electronic survey system Qualtrics^2^. This data gathering was conducted in the second and third weeks of March 2021.

### Assessment Procedure

Participants clicked a link to the survey system. The first page of the system contained basic information about the content of the survey, legal issues, and data storage, it also asked participants for informed consent by clicking the button to the next page. After questions on demographic variables, the operationalisations of the general and behaviour-specific psychological factors, and the indicators of behaviours were presented using various question formats. The last page thanked participants.

### Measurements

#### Background Variables

The measurement started with assessing gender, level of education, age, whether the participants had been tested for COVID-19 (yes/no), and what the test result was.

#### The General Determinants

Susceptibility was assessed with the question: “How high is the chance (%) that in the coming 3 months you will get ill from the coronavirus?”. A horizontal slide bar was presented that could be set to indicate a percentage from 0 to 100.

Seriousness was assessed with the question: “How bad is it to get contaminated and get ill because of the coronavirus?”. The answering options were: “Not bad at all” (1); “A little bad” (2); “Just bad” (3); “Very bad” (4); “Awfully bad” (5).

Fear was assessed with the question: “During the past week, did you feel afraid to get contaminated and get ill because of the coronavirus?”. The answering options were: “Never” (1); “Seldom” (2); “Sometimes” (3); “Regularly” (4); “Often” (5); “Very often” (6); “Always” (7).

Perceived control was assessed with the question: “Are you able to exert influence yourself on whether you will get contaminated?”. The answering options were: “I have no influence at all” (1); “I have little influence” (2); “I do have some influence” (3); “I have substantial influence” (4); “I can influence it myself completely” (5).

#### Behaviour-Specific Determinants and Behaviours

##### Prevention Behaviour

Whether people “tried to adhere to the basic corona behavioural guidelines (such as keeping distance, hand washing, wear a face mask, not visiting, etc.),” could be indicated with the answering options: “I don’t try at all” (0); “I try somewhat” (1); “I try” (2); “I try as much as possible” (3). Thus, this measurement only used a single item referring to all recommended behaviours. This item was the indicator of prevention behaviour. No behaviour-specific determinants of this behaviour were assessed in this data gathering.

##### Getting Tested

Three behaviour-specific determinants of getting tested were assessed. The first measure was: “How reliable do you think the common CPR test is?”. The answer could be given on a 5-point scale, from “Not reliable at all” (1) to “Very reliable” (5).

The second measure was: “When you would be tested with the CPR test, and it is positive, how high is the chance (%) that you still do not have COVID-19?”. A horizontal slide bar was presented that could be set to indicate a percentage from 0 to 100.

The third measure assessed social influence with the item: “Do you think it is your social duty to get tested when you have complaints?”. The answer could be given on a 5-point scale, from “Not at all” (1) to “Very strong” (5).

The intention to get tested was assessed with the question: “When for 2 days you cough, have a running nose, and you sneeze, will you get tested?”. The answer could be given on a 5-point scale, from “Certainly not” (1) to “Certainly” (5). This item was the indicator of testing behaviour.

##### Getting Vaccinated

Two behaviour-specific determinants of getting vaccinated were assessed. The first measure assessed perceived effectiveness: “In how many of 100 people, do you think, will the vaccination really protect against COVID-19?”. A horizontal slide bar was presented that could be set to indicate the number of people from 0 to 100.

The second measure assessed social influence with the item: “Do you think it is your social duty to get vaccinated?”. The answer could be given on a 5-point scale, from “Not at all” (1) to “Very strong” (5).

The intention to get vaccinated was assessed with a composite of two questions: “How strongly do you desire to get vaccinated against COVID-19 in 2021?”. The answer could be given on a 5-point scale, from “Not desire at all” (1) to “Very strong desire” (5); and “How certain are you that in 2021 you will get vaccinated against COVID-19?”. The answer could be given on a 5-point scale, from “Certainly not” (1) to “Certainly” (5). The mean item score was computed to be the scale score (sample 1 *r* = 0.29; *p* < 0.001; sample 2 *r* = 0.86; *p* < 0.001). This two-item scale was the indicator of vaccination behaviour.

#### Conspiracy Beliefs

Endorsement of conspiracy beliefs was assessed with a five-item scale. The items were based on observations in the general and social media, the literature, and on an earlier survey (van der Meulen, 2020, October 31). Agreement with each of the following five statements was assessed: “The [Dutch] government consciously holds back information on covid-19”; “The RIVM [Dutch public health organisation] is not honest about covid-19 vaccinations”; “The coronavirus has been developed and spread deliberately”; “The media coverage about covid-19 is controlled by the government”; “The seriousness of the covid-19 epidemic has been consciously exaggerated by the OMT [The Dutch health authorities]”. The answering options were: “Completely disagree” (1); “Disagree somewhat” (2); “Not disagree/not agree (3); “Agree somewhat” (4); “Completely agree” (5). The average score was used as the scale score (sample 1 α = 0.86; sample 2 α = 0.86). The higher the score, the stronger participants agree that the government and the government organizations have covert motivations that may indicate a conspiracy.

### The Mediation Analyses

The relationship of CBc with prevention behaviour, testing self-report and intention, and vaccination intention were expected to be mediated by the general and behaviour-specific determinants of behaviour. Behaviour-specific determinants were not assessed for prevention behaviour (by mistake). Mediation was tested using model 4 of the SPSS 25 PROCESS module of Hayes^3^, with 5,000 bootstraps. Mediation was considered to be significant when the 95% CI of the coefficient of the potential mediator did not include zero. Since there are large differences between the scales, the variables were all transformed into *z*-scores. Complex mediational relationships may exist between general and behaviour-specific determinants in their relationship with the dependent variables. Therefore, general and behaviour-specific determinants were tested for mediation separately. Firstly, mediation by the general determinants was tested for all four indicators of behaviours. Secondly, for self-reported testing and intention, and vaccination intention, the additional behaviour-specific determinants were tested as potential mediators. Thirdly, the combined general and behaviour-specific determinants were tested.

## Results

### Participant Characteristics

Sample 1: Among the 1,004 people in this sample, 50.4% were women, 65.3% were classified as having a high level of education, 49.4% reported to have been tested for COVID-19, of whom 13.5% (*n* = 67) was tested positive. The mean age was 50.5 (*SD* = 17.4). The mean scores on conspiracy beliefs were 2.38 (*SD* = 1.12), while 26.5% of the participants scored above the neutral midpoint of 3. Furthermore, 57% reported having been tested for COVID-19. With regard to vaccination intention, data from 94 participants were missing as they had already been vaccinated.

Sample 2: Among the 159 people in this sample, 68% were women, 79% were classified as having a high level of education, 49% reported to have been tested for COVID-19, of whom 6% (*n* = 5) was tested positive. The mean age was 47.6 (*SD* = 15.8). The mean score on conspiracy beliefs was 2.11 (*SD* = 1.05), while 18.2% of the participants scored above the neutral midpoint of 3. Furthermore, 51% reported having been tested for COVID-19. With regard to vaccination intention, data from 10 participants were missing as they had already been vaccinated. The descriptives of the psychological and behavioural variables can be found in the Appendix.

### Univariate Relations

Table 1 presents the univariate relations (Spearman correlations) between CBc, the indicators of the three behaviours, the general determinants, and the behaviour-specific determinants, in the two samples. Firstly, in both samples CBc were significantly and negatively related to three of the four indicators of the behaviours; the correlations with Testing self-report were not significant.

**TABLE 1:** Spearman correlations among the variables in the two samples.

**Sample 2\Sample 1**	**1**	**2**	**3**	**4**	**5**	**6**	**7**	**8**	**9**	**10**	**11**	**12**	**13**	**14**
(1) Conspiracy beliefs		–0.33	–0.01ns	–0.29	–0.48	–0.07	–0.22	–0.17	–0.24	–0.45	0.27	–0.36	–0.42	–0.45
(2) Prevention	–0.27		–0.07	0.29	0.43	0.02ns	0.38	0.24	0.27	0.28	–0.11	0.35	0.26	0.40
(3) Test self-report	–0.08ns	–09ns		0.19	–0.02ns	0.15	–0.04ns	0.03ns	–0.03ns	0.10	–0.07	0.20	–0.02ns	–0.03ns
(4) Testing intention	–0.19	0.16	0.17		0.38	0.14	0.20	0.18	0.2	0.34	–0.19	0.65	0.25	0.33
(5) Vaccination intention^1^	–0.32	0.15ns	–04ns	–0.00ns		0.08	0.41	0.30	0.26	0.46	–0.21	0.44	0.51	0.75
(6) Susceptibility	0.5ns	0.12ns	–0.06ns	0.04ns	0.02ns		0.07	0.36	–0.19	0.08	0.05ns	0.13	0.00ns	0.04ns
(7) Seriousness	–0.19	0.44	–0.03ns	0.05ns	0.33	0.15ns		0.44	0.22	0.23	–0.07	0.32	0.20	0.34
(8) Fear	–0.12ns	0.35	0.08ns	0.09ns	0.08ns	0.31	0.40		0.01ns	0.17	0.02ns	0.23	0.11	0.24
(9) Control	–0.32	0.29	–0.04ns	0.31	0.00ns	–0.19	0.01ns	0.04ns		0.20	–0.13	0.23	0.20	0.22
(10) Test reliability	–0.57	0.25	0.09ns	0.26	0.33	0.01ns	0.18	0.14ns	0.26		–0.47	0.40	0.44	0.41
(11) Fault positive	0.42	–0.13	–0.07ns	–0.17	–0.24	0.00ns	–0.03ns	–0.17	–0.19	–0.60		–0.25	–0.27	–0.16
(12) Social duty testing	–0.33	0.40	0.30	0.45	0.25	0.14ns	0.22	0.28	0.27	0.45	–0.39		0.31	0.45
(13) Effectiveness	–0.35	0.09ns	0.03ns	0.13ns	0.23	0.08ns	0.01ns	0.04ns	0.17	0.48	–0.37	0.25		0.46
(14) Social duty vaccination	–0.56	0.35	0.21	0.26	0.31	0.04ns	0.24	0.33	0.19	0.44	–0.37	0.53	0.36	

*Correlations with the addition “ns” are not significant. The correlations in the upper-right half are from sample 1 (*N* = 1,004). The correlations in the lower-left half are from sample 2 (*N* = 159).*

*^1^*n* = 910 and 149 in samples 1 and 2, respectively, due to missing data (these participants had already been vaccinated).*

Secondly, concerning the general determinants, the correlations of CBc with seriousness and control were significant and negative in both samples. In sample 2, the correlations with susceptibility and fear were not significant (in sample 1 these correlations were the smallest).

Thirdly, concerning the behaviour-specific determinants, the correlations were all significant in both samples. Four of the five correlations were negative, but as expected, the correlations with “false positive rates of testing” were positive. CBc were especially strongly related to both items on social duty, signalling perceiving testing and vaccination less as a social duty as participants more strongly endorsed CBc.

In both samples, the smallest or most non-significant correlations with all other variables concerned those with susceptibility and testing self-report. Furthermore, all correlations were in expected directions. Besides the differences in the statistical power of the tests in both samples, the pattern of results in both samples was very similar.

### Factor Analyses

To check the theoretical assumption that behaviour-specific determinants can be distinguished from general determinants, factor analyses (with oblique rotation) were conducted on the nine items that assessed these determinants in both samples. The pattern matrix showed that the five items on the behaviour-specific determinants clearly loaded on one factor in both samples (see Appendix). The four general determinants appeared in two factors in both samples (clustering susceptibility with control, and seriousness with fear). These results mean that the expected structure was partly verified: the behaviour-specific determinants can be distinguished from the general determinants. Therefore, three sets of mediation analyses were conducted: The mediators being the general determinants, the behaviour-specific determinants, or the combined determinants.

### Mediation by General Determinants

#### Prevention Behaviours

In sample 1, the model with prevention behaviour as the dependent variable, CBc as an independent variable, and susceptibility, seriousness, fear, and control as mediators, explained 28% in the variance of prevention behaviour. CBc had a significant direct relationship with prevention behaviour (–0.27, *p* < 0.0001). Significant unstandardised indirect effects were present with regard to seriousness [–0.07 (CI –0.096 to –0.048)], fear [–0.011 (CI –0.02 to –0.003)], and control, [–0.036 (CI –0.058 to –0.017)]. Among the relationship of CBs with prevention behaviour, 30% was mediated. This test showed partial mediation.

**FIGURE 1:**
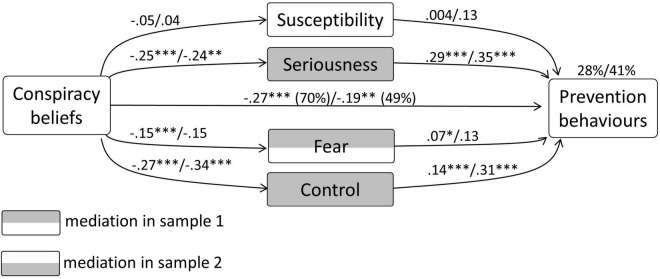
Results from the mediation analysis regarding Conspiracy Beliefs concerning corona (CBc), prevention behaviours, and the general psychological determinants.

In sample 2 this model explained 41% in the variance of prevention behaviour. CBc had a significant direct relationship with prevention behaviour, –0.19, *p* < 0.01. Significant unstandardized indirect effects were present with regard to seriousness [–0.082 (CI –0.16 to –0.018] and control [–0.10 (CI –0.20 to –0.04)]. Regarding the relationship of CBc with prevention behaviour, 51% was mediated. This test showed partial mediation. The diagram concerning the mediation model in both samples is depicted in Figure 1.

#### Testing

Two indicators of testing behaviour were used as dependent variables: Test self-report (yes/no), and the intention to get tested. The models contained CBc as an independent variable, and susceptibility, seriousness, fear, and control as mediators.

With regard to testing self-report the explained variances of the models in both samples were low (sample 1: 3.9%; sample 2: 5.6%). In both samples, CBc had no significant direct relationship with testing, and no mediation was found. Because of the low explained variances in both samples no diagram is presented.

With regard to testing intention, in sample 1 the model explained 19% in the variance. CBc had a significant direct relationship with testing intention (–0.29, *p* < 0.0001). Significant unstandardized indirect effects were present with regard to seriousness [–0.022 (CI –0.04 to –0.004)], fear [–0.011 (CI –0.02 to –0.001)], and control [–0.037 (CI –0.059 to –0.018)]. Regarding the relationship of CBc with testing intention, 21% was mediated. This test showed partial mediation.

**FIGURE 2:**
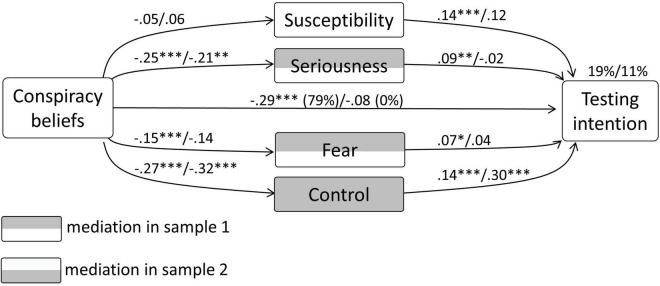
Results from the mediation analysis concerning CBc, testing intention, and the general psychological determinants.

In sample 2 this model explained 11% in the variance of testing intention. CBc had no significant direct relationship with testing intention, *p* > 0.37. Significant unstandardized indirect effects were present only with regard to control [–0.096 (CI –0.18 to –0.029)]. This test showed complete mediation. The diagram concerning the mediation model in both samples is depicted in Figure 2.

#### Vaccination Intention

In sample 1, the model with vaccination intention as the dependent variable, CBc as an independent variable, and susceptibility, seriousness, fear, and control as mediators, explained 41% in the variance of vaccination intention. CBc had a significant direct relationship with vaccination intention (–0.44, *p* < 0.0001). Significant unstandardised indirect effects were present with regard to seriousness [–0.063 (CI –0.086 to –0.042)], fear [–0.013 (CI –0.025 to –0.004)], and control [–0.026 (CI –0.045 to –0.01)]. Regarding the relationship of CBc with vaccination intention, 19% was mediated. This test showed partial mediation.

**FIGURE 3:**
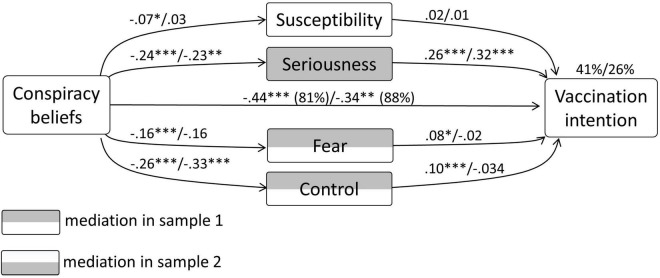
Results from the mediation analysis concerning CBc, vaccination intention, and the general psychological determinants.

In sample 2 this model also explained 26% in the variance of vaccination intention. CBc had a significant direct relationship with vaccination intention (–0.34, *p* < 0.0001). A significant unstandardized indirect effect was present with regard to seriousness only [–0.072 (CI –0.15 to –0.01)]. Of the relationship of CBc with vaccination intention, 22% was mediated. This test showed partial mediation. The diagram concerning the mediation model in both samples is depicted in Figure 3. A summary of all coefficients and their confidence intervals can be found in the Appendix.

### Behaviour-Specific Determinants

#### Testing

The models contained test self-report and testing intention as dependent variables, CBc as an independent variable, and test reliability, false positives, and social duty for testing as mediators.

With regard to test self-report as the dependent variable, the explained variance of the models in both samples was low again (sample 1: 5.7%; sample 2: 9.7%). Only in sample 1, CBc had a significant direct relationship with testing [0.21 (CI 0.06–0.36)]. In both samples social duty mediated the relationship significantly: sample 1: –0.19 (CI –0.27 to –0.12); sample 2: –0.30 (CI –0.63 to –0.095). Because of the low explained variances in both samples no diagram is presented.

With regard to testing intention as the dependent variable, in sample 1 the model explained 53% in the variance. CBc had no significant direct relationship with testing intention, *p* > 0.29. Significant unstandardized indirect effects were present with regard to test reliability [–0.043 (CI –0.077 to –0.012)] and social duty for testing [–0.29 (CI –0.35 to –0.24)]. This test showed complete mediation.

**FIGURE 4:**
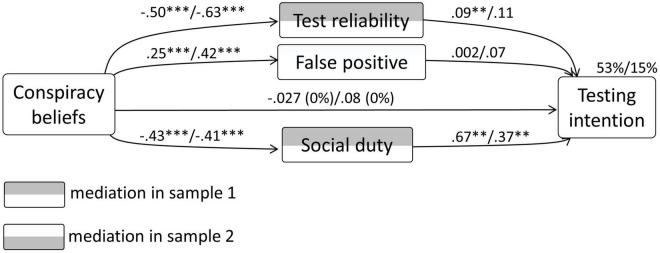
Results from the mediation analysis concerning CBc, testing intention, and the behaviour-specific psychological determinants.

In sample 2, the model explained 15% in the variance of testing intention. CBc had no significant direct relationship with testing intention, *p* > 0.78. No significant unstandardized indirect effects were present. This test showed no mediation. The diagram concerning the mediation model in both samples is depicted in Figure 4.

#### Vaccination Intention

In sample 1, the model with vaccination intention as the dependent variable, CBc as an independent variable, and effectiveness and social duty for vaccination as mediators, explained 73% in the variance of vaccination intention. CBc had a significant direct relationship with vaccination intention (–0.11, *p* < 0.0001). Significant unstandardised indirect effects were present with regard to effectiveness [–0.091 (CI –0.12 to –0.063)] and social duty for vaccination [–0.34 (CI –0.40 to –0.29)]. Regarding the relationship of CBc with vaccination intention, 80% was mediated. This test showed partial mediation.

**FIGURE 5:**
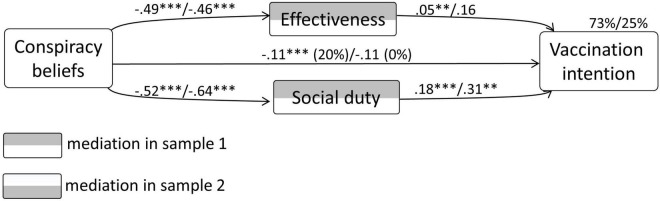
Results from the mediation analysis concerning CBc, vaccination intention, and the behaviour-specific psychological determinants.

In sample 2, the model explained 25% in the variance of vaccination intention. CBc had no significant direct relationship with vaccination intention, *p* > 0.23. No significant unstandardized indirect effects were present either. This test showed no mediation. The diagram concerning the mediation model in both samples is depicted in Figure 5. A summary of all coefficients and their confidence intervals can be found in the Appendix.

### The Combined General and Behaviour-Specific Determinants

Because of the overlap, no diagrams were presented of these analyses. The main mediation results were reported here, and all coefficients and their confidence intervals could be found in the Appendix.

#### Testing

The models contained test self-report and testing intention as dependent variables, CBc as an independent variable, and all four general and the three behaviour-specific determinants as mediators.

With regard to test self-report as the dependent variable in sample 1, the explained variance was only 9.7%. The direct relationship of CBc with testing was significant (0.18, *p* < 0.05). Significant unstandardized indirect effects were present with regard to seriousness [0.06 (CI 0.018–0.11)] and social duty for testing [–0.21 (CI –0.30 to –0.13)]. Thus, the mediation was partial.

With regard to test self-report as the dependent variable in sample 2, the explained variance was 19%. The direct relationship of CBc with testing was not significant (*p* > 0.52). Significant unstandardized indirect effects were present with regard to control [0.20 (CI 0.059–0.45)] and social duty for testing [–0.44 (CI –0.95 to –0.16)]. This test showed complete mediation.

With regard to testing intention as the dependent variable, in sample 1 the model explained 54% in the variance. CBc had no significant direct relationship with testing intention, *p* > 0.34. Significant unstandardized indirect effects were present with regard to test reliability [–0.042 (CI –0.075 to –0.011)] and social duty for testing [–0.29 (CI –0.35 to –0.24)]. This test showed complete mediation.

In sample 2, the model explained 21% in the variance of testing intention. CBc had no significant direct relationship with testing intention, *p* > 0.43. Only with regard to control the unstandardized indirect effect was significant [–0.082 (CI –0.24 to –0.06)]. This test showed complete mediation.

#### Vaccination Intention

In sample 1, the model with vaccination intention as the dependent variable, CBc as independent variable, and all four general and the two behaviour-specific determinants as mediators, explained 75% in the variance of vaccination intention. CBc had a significant direct relationship with vaccination intention, –0.10, *p* < 0.0001. Significant unstandardised indirect effects were present with regard to seriousness [–0.03 (CI –0.038 to –0.013)], control [–0.012 (CI –0.022 to –0.003)], effectiveness [–0.087 (CI –0.12 to –0.06)], and social duty for vaccination [–0.32 (CI –0.37 to –0.27)]. Regarding the relationship of CBc with vaccination intention, 82% was mediated. This test showed partial mediation.

In sample 2, this model explained 31% in the variance of vaccination intention. CBc had no significant direct relationship with vaccination intention, *p* > 0.23. A significant unstandardized indirect effect was present only for seriousness [–0.057 (CI –0.013 to –0.0003)] This test showed complete mediation.

## Discussion

The aim of this study was to unravel how endorsement of CBc might influence corona-related behaviours, in the context of media information on COVID-19 and the corona-crisis in the Netherlands. It was tested whether the relationship between CBc and prevention behaviours, test self-report, the intention to get tested, and the intention to get vaccinated, was mediated by the general and behaviour-specific determinants of the behaviours.

The preparatory analyses showed that the correlations within both samples were similar. CBc were related to the other variables, mostly in the expected directions: The stronger participants endorsed CBc, the less serious they found COVID-19, the less fear they experienced for getting contaminated and ill, the less control they perceived over getting contaminated, the less reliable they estimated the PCR test, the higher they estimated the number of false-positive tests, the lower they estimated the effectiveness of vaccination, and the less they felt that testing and vaccination was their social duty. These results are congruent with the notion that those who endorse CBc reject the information from the general media and from others who are informed by those media.

The correlations of CBc with social duty (with regard to testing and vaccination) ranged from –0.33 to –0.56. This is consistent with earlier findings of Pummerer et al. (2021) that showed that those who endorse CBc show lower institutional trust, lower support of governmental regulations, less adoption of physical distancing, and less social engagement (also see O’Connell et al., 2021). The rejection of social responsibility may be seen as a logical consequence of perceiving COVID-19 as less serious; when there is no problem there is no necessity to fulfil a social duty.

The negative correlations between CBc and perceived control are consistent with earlier findings that CT is related to feelings of powerlessness (Abalakina-Paap et al., 1999). However, the present measure of control was specific to controlling the chance of getting contaminated. Surprisingly, it was positively related to getting tested, while this is not a behaviour that effectively provides control over contamination. The measure seems to have captured more a sense of general necessity or urgency to do something; it may be confounded by feelings of general necessity or urgency. This is consistent with the negative correlations between CBc and perceived control: The stronger the endorsement of CBc, the lower the control scores, possibly indicating a lower urgency.

Conspiracy Beliefs concerning corona were hardly related to estimates of personal vulnerability to get contaminated. This might mean that CBc do not downplay media information about the chances of contamination, in contrast to, for example, media information on seriousness. On the other hand, estimates of one’s susceptibility may be unstable, reflecting their momentary use in emotion-regulation as conceptualised in unrealistic optimism (Weinstein, 2003), which undermines their predictive nature (Dijkstra and Buunk, 2008).

The correlations of test self-report were mostly non-significant, while the correlations with testing intention were positive but small. This may mean that a self-report of having been tested is hardly determined by psychological factors. Indeed, the lack of these relationships suggests that getting tested is more strongly related to other determinants, such as being contaminated and the manifestation of symptoms that might be interpreted by the individual to indicate COVID-19.

Together, these simple correlations, consistent in both samples, draw a revealing picture of how people who endorse CBc experience the present corona crisis. While people who do not endorse CBs seem to think largely in line with the information from the general media, people who do endorse CBc seem to develop in the opposite direction. However, it is not only about their thinking, but it is also about their behaviour that can influence others and can also influence the pandemic. The mediation analyses were meant to provide insight into how the lowered levels of corona-related behaviours can be caused by CBc. The results, indeed, showed mediation by psychological determinants.

Concerning the general determinants, the results were rather consistent: In the larger sample, perceived seriousness of COVID-19, the experience of fear, and perceived control mediated the relationship between CBc and three of the four indicators of behaviour. In the smaller sample, these findings were partly replicated, with regard to seriousness and control. In both samples, the analyses with regard to the dichotomous variable, test self-report (yes/no), revealed no mediation at all by the general determinants. The low explained variances suggest that the general determinants and CBc were hardly related to being tested.

Concerning the behaviour-specific determinants, mediation was mainly found in the larger sample. We can assume that this is caused by lower statistical power in the smaller sample (Kenny, 2021, August 2) or by the selection of participants. In the larger sample, test reliability and social duty to test, mediated the relationship with testing intention, while effectiveness and social duty to vaccinate mediated the relationship with vaccination intention. With regard to testing self-report (yes/no), in both samples, social duty was a significant mediator. Although the variance that was explained by the whole model was low, it seems that having been tested was predicted by a behaviour-specific determinant, rather than by general determinants. This is in line with the principle of the TPB that specific behaviours are primarily caused by specific beliefs (Ajzen, 1991). It also stresses the importance of this social or personal norm in determining corona-related behaviours.

Conspiracy Beliefs concerning corona still had a direct relationship with the dependent variable in several tests. In sample 1, in three of the four tests with the general determinants, and two of the three tests with the behaviour-specific determinants this was the case. From a theoretical point of view, this cannot be explained. The theory does propose a sequence between the determinants, implying another type of mediation: The general determinants will lead to the behaviour-specific determinants, that will lead to behaviour (this specific mediation is not the focus of this study). It might be argued that analysing both types of determinants separately, left room for CBc to relate directly to the indicators of the three corona-related behaviours. However, the combined analyses still showed this direct relationship in two of the three analyses. This suggests that not all determinants were assessed, or that the operationalisations of the determinant measures were not optimal.

Besides this unexplained finding, in most of the ten mediation tests, complete or partial mediation by determinants was found (sample 1: nine of the ten; sample 2: seven of the ten). The results on mediation are explained using two theoretical angles. Firstly, CBc can be expected to inspire “major premises” (Kruglanski et al., 2006) that lead to rejection of information from general media. In a general media landscape in which COVID-19 is presented as a serious disease and vaccination as effective; this leads to rejecting the seriousness of COVID-19 and the effectiveness of vaccination. The data suggest that stronger endorsement of CBc leads to stronger rejection, for example, by stronger downplaying the seriousness and effectiveness. Secondly, these perceptions comprise the general and specific determinants of corona-related behaviours, that determine behaviour as conceptualised in models of psychological determinants, EPPM (Witte, 1992), TPB, and SCT (Hagger et al., 2020).

Although this perspective on how CBc leads to less engagement in corona-related behaviours has a theoretical foundation, other pathways are possible. That is, one assumption in the present study was that people in the Netherlands are all exposed to the general media; no variance in exposure was assessed. In addition, no measurements of exposure, for example, to social media that spread critical information on the pandemic or even misinformation, were assessed. As Romer and Jamieson (2020) suggest, these latter media may propagate conspiracy beliefs, and communicate that COVID-19 is not serious, etc. Thus, while the present position is that CBc leads people to interpret the information from general media reactively, thereby leading them to reject their message that COVID-19 is serious, etc., it is also possible that for different reasons people get exposed to the more critical social media, from which they learn about conspiracy, and at the same time learn that COVID-19 is not serious, etc. Future studies may contrast both positions and reveal whether they are competing or complementary.

This study had some relevant limitations that should be borne in mind when interpreting the results. Most measures were one-item measures. This was to address all three behaviours but at the same time to avoid a long questionnaire. These simple measurements can be unreliable (Sarstedt and Wilczynski, 2009), although psychology has several examples of valid single-item scales (e.g., Bergkvist and Rossiter, 2007; Williams and Smith, 2016). Furthermore, the smaller sample comprised a selection of participants, largely female and highly educated, who self-selected for an earlier similar (but not the same) questionnaire 1 year earlier. Still, the results in both samples, certainly with regard to the correlations, were rather similar, which provides some confidence that the effect of the selection was limited. Another limitation is that the behaviours of testing and vaccination were only assessed with their preceding intentions. Although it can be expected that these measures do predict actual behaviours (Sheeran, 2002), the present study did not show this. One more pity detail is that, mistakenly, no behaviour-specific determinants of prevention behaviours were assessed, only general ones. Also, one obvious limitation is the cross-sectional design: Although the conceptualised mechanisms of mediation are plausible, the present study cannot prove causality. Although the results in both samples were similar, the differences in mediation may be caused by differences in statistical power, with the larger sample also showing smaller effects that are not detected in the smaller sample.

Despite these shortcomings, the results show some meaningful robust patterns. Although mediation has been studied earlier to explain the effects of CT on health behaviours (e.g., Jolley and Douglas, 2014b), no earlier studies addressed a full model of the psychological mechanisms involved in the relation between CBc and the (indicators of) behaviours. The results can inspire (experimental) studies to further address the processes with which CBc influences human thinking and behaviour in a collective health crisis in which the general media have an important function.

## Data Availability Statement

The raw data supporting the conclusions of this article will be made available by the authors, without undue reservation.

## Ethics Statement

The studies involving human participants were reviewed and approved by the Ethical Committee Psychology (ECP) of the Faculty of Behavioural and Social Sciences. The patients/participants provided their written informed consent to participate in this study.

## Author Contributions

AD has conducted all tasks concerning the design of the study, the statistical analysis, and writing the manuscript.

## Conflict of Interest

The author declares that the research was conducted in the absence of any commercial or financial relationships that could be construed as a potential conflict of interest.

## Publisher’s Note

All claims expressed in this article are solely those of the authors and do not necessarily represent those of their affiliated organizations, or those of the publisher, the editors and the reviewers. Any product that may be evaluated in this article, or claim that may be made by its manufacturer, is not guaranteed or endorsed by the publisher.

## Footnotes

1
www.enigmaresearch.nl

2
www.qualtrics.com

3
afhayes.com
