# Supplementary material for: A Mediation Model on How Conspiracy Beliefs Concerning the Corona-Crisis Are Related to Corona-Related Behaviours
Source: Front Psychol. 2021 Nov 22;12:740888. doi: 10.3389/fpsyg.2021.740888 (PMC8645847; doi:10.3389/fpsyg.2021.740888)
Supplement: Supplementary file 1 [file Data_Sheet_1.docx]

Appendix 1. Means (and SD) and comparison of the mean scores in both samples.

|  | Sample 1  (N=1004) | Sample 2  (N=159) | p-value |
| --- | --- | --- | --- |
| 1. Conspiracy beliefs | 2.38 (1.12) | 2.11 (1.06) | .006 |
| 2. Prevention behaviours | 3.49 (0.78) | 3.61 (0.75) | .08 |
| 3. Test self-report, yes | 49% | 52% | .51 |
| 4. Testing intention | 4.17 (1.14) | 3.61 (1.07) | <.001 |
| 5. Vaccination intention^1^ | 3.99 (1.34) | 3.55 1.04) | <.001 |
| 6. Susceptibility | 28.4 (21.8) | 29.5 (18.5) | .55 |
| 7. Seriousness | 3.1 (1.00) | 3.09 (1.03) | .85 |
| 8. Fear | 2.53 (1.24) | 2.51 (1.15) | .82 |
| 9. Control | 3.26 (0.88) | 3.42 (0.79) | .04 |
| 10. Test reliability | 3.77 (1.05) | 3.89 (1.02) | .16 |
| 11. Fault positive | 27.1 (27.7) | 22.9 (25.9) | .07 |
| 12. Social duty testing | 4.17 (1.13) | 4.31 (1.13) | .13 |
| 13. Effectiveness | 73.2 (22.9) | 75.5 (19.7) | .25 |
| 14. Social duty vaccination | 3.90 (1.42) | 4.15 (1.30) | .04 |

^1^ n=910 and 149 in sample 1 and 2, respectively, due to missing data

(these participants had already been vaccinated).

Appendix 2. Factor loadings (pattern matrix) of the general and behaviour-specific determinants.

|  | Sample 1 | | | Sample 2 | | |
| --- | --- | --- | --- | --- | --- | --- |
| Factors | 1 | 2 | 3 | 1 | 2 | 3 |
| 1. Susceptibility |  |  | -.76 |  |  | -.79 |
| 2. Seriousness |  | .75 |  |  | .86 |  |
| 3. Fear |  | .76 |  |  | .73 |  |
| 4. Control |  |  | .72 |  |  | .69 |
| 5. Test reliability | .83 |  |  | .85 |  |  |
| 6. Fault positive | -.67 | .42 |  | -.73 |  |  |
| 7. Social duty testing | .66 |  |  | .67 |  |  |
| 8. Effectiveness | .74 |  |  | .79 |  |  |
| 9. Social duty vaccination | .64 |  |  | .65 |  |  |

The loadings in the empty cells are all below .42. Oblique rotation was applied.

Appendix 3. Mediation effects and their 95% bootstrapping confidence intervals of the general determinants.

|  | **Sample 1** | | | **Sample 2** | | |
| --- | --- | --- | --- | --- | --- | --- |
| Mediator | Effect | CI Lower | CI Upper | Effect | CI Lower | CI Upper |
| **Prevention** | | | | | | |
| Susceptibility | -,0002 | -,0040 | ,0035 | ,0056 | -,0164 | ,0351 |
| Seriousness | **-,0708** | **-,0949** | **-,0478** | **-,0817** | **-,1575** | **-,0186** |
| Fear | **-,0107** | **-,0202** | **-,0028** | -,0199 | -,0475 | ,0022 |
| Control | **-,0362** | **-,0586** | **-,0177** | **-,1033** | **-,1963** | **-,0341** |
| **Test self-report** | | | | | | |
| Susceptibility | -,0154 | -,0403 | ,0065 | -,0096 | -,0781 | ,0356 |
| Seriousness | ,0304 | -,0052 | ,0717 | ,0415 | -,0430 | ,1834 |
| Fear | -,0026 | -,0255 | ,0210 | -,0360 | -,1319 | ,0226 |
| Control | -,0068 | -,0448 | ,0297 | ,1120 | -,0061 | ,2708 |
| **Testing intention** | | | | | | |
| Susceptibility | -,0061 | -,0159 | ,0028 | ,0078 | -,0155 | ,0439 |
| Seriousness | **-,0221** | **-,0411** | **-,0044** | ,0045 | -,0295 | ,0494 |
| Fear | **-,0105** | **-,0209** | **-,0011** | -,0055 | -,0330 | ,0178 |
| Control | **-,0365** | **-,0599** | **-,0177** | **-,0974** | **-,1819** | **-,0299** |
| **Vaccination intention** | | | | | | |
| Susceptibility | -,0013 | -,0065 | ,0026 | ,0002 | -,0204 | ,0199 |
| Seriousness | **-,0631** | **-,0863** | **-,0418** | **-,0724** | **-,1520** | **-,0071** |
| Fear | **-,0131** | **-,0248** | **-,0037** | ,0025 | -,0319 | ,0395 |
| Control | **-,0262** | **-,0449** | **-,0102** | ,0108 | -,0445 | ,0748 |

Coefficients in bold refer to significant mediation.

Appendix 4. Mediation effects and their 95% bootstrapping confidence intervals of the behaviour-specific determinants.

|  | **Sample 1** | | | **Sample 2** | | |
| --- | --- | --- | --- | --- | --- | --- |
| Mediator | Effect | CI Lower | CI Upper | Effect | CI Lower | CI Upper |
| **Test self-report** | | | | | | |
| Test reliability | -,0028 | -,0403 | ,0322 | ,1194 | -,2061 | ,5167 |
| Fault positive | -,0535 | -,1419 | ,0299 | ,0286 | -,1630 | ,2273 |
| Social duty testing | **-,1874** | **-,2723** | **-,1168** | **-,2986** | **-,6293** | **-,0950** |
| **Testing intention** | | | | | | |
| Test reliability | **-,0432** | **-,0765** | **-,0116** | -,0701 | -,2461 | ,0804 |
| Fault positive | ,0005 | -,0123 | ,0126 | ,0282 | -,0612 | ,1411 |
| Social duty testing | **-,2912** | **-,3477** | **-,2380** | -,1487 | -,3380 | ,0007 |
| **Vaccination intention** | | | | | | |
| Effectiveness | **-,0905** | **-,1210** | **-,0627** | -,0719 | -,1828 | ,0134 |
| Social duty vaccination | **-,3418** | **-,3954** | **-,2913** | -,1958 | -,3906 | ,0049 |

Coefficients in bold refer to significant mediation.

Appendix 5. Mediation effects and their 95% bootstrapping confidence intervals of the combined general determinants and the behaviour-specific determinants.

|  | **Sample 1** | | | **Sample 2** | | |
| --- | --- | --- | --- | --- | --- | --- |
| Mediator | Effect | CI Lower | CI Upper | Effect | CI Lower | CI Upper |
| **Test self-report** | | | | | | |
| Susceptibility | -0,0135 | -0,0357 | 0,006 | -0,0131 | -0,1043 | 0,0566 |
| Seriousness | **0,0576** | **0,0182** | **0,1048** | 0,075 | -0,0129 | 0,2331 |
| Fear | 0,0027 | -0,0204 | 0,0285 | -0,0222 | -0,111 | 0,0455 |
| Control | 0,0122 | -0,026 | 0,0523 | **0,2035** | **0,059** | **0,4516** |
| Test reliability | -0,0588 | -0,1497 | 0,0292 | 0,1061 | -0,2632 | 0,5425 |
| Fault positive | -0,0074 | -0,0465 | 0,0298 | 0,0517 | -0,1708 | 0,2857 |
| Social duty testing | **-0,2065** | **-0,2983** | **-0,1318** | **-0,4426** | **-0,9509** | **-0,1571** |
| **Testing intention** | | | | | | |
| Susceptibility | -0,0027 | -0,008 | 0,0011 | 0,0035 | -0,0132 | 0,0296 |
| Seriousness | 0,0121 | -0,0008 | 0,0264 | 0,0207 | -0,0095 | 0,0672 |
| Fear | -0,0026 | -0,0102 | 0,0046 | -0,0035 | -0,0286 | 0,0219 |
| Control | -0,0125 | -0,0271 | 0,0000 | **-0,0817** | **-0,1648** | **-0,0137** |
| Test reliability | **-0,0418** | **-0,0746** | **-0,0105** | -0,0819 | -0,2426 | 0,0638 |
| Fault positive | -0,0004 | -0,0133 | 0,0114 | 0,0319 | -0,0513 | 0,1354 |
| Social duty testing | **-0,2887** | **-0,3459** | **-0,2359** | -0,1319 | -0,3168 | 0,012 |
| **Vaccination intention** | | | | | | |
| Susceptibility | 0,0001 | -0,0026 | 0,003 | 0,000 | -0,0209 | 0,0219 |
| Seriousness | **-0,0246** | **-0,0375** | **-0,0133** | **-0,0574** | **-0,1318** | **-0,0003** |
| Fear | -0,0045 | -0,0116 | 0,002 | 0,0109 | -0,0204 | 0,0547 |
| Control | **-0,0121** | **-0,0221** | **-0,003** | 0,0158 | -0,0318 | 0,0706 |
| Effectiveness | **-0,087** | **-0,1165** | **-0,06** | -0,0763 | -0,1724 | 0,0055 |
| Social duty vaccination | **-0,3182** | **-0,3701** | **-0,2676** | -0,1569 | -0,3519 | 0,0453 |

Coefficients in bold refer to significant mediation.
